# Adverse Events during Vitrectomy under Adequacy of Anesthesia—An Additional Report

**DOI:** 10.3390/jcm10184172

**Published:** 2021-09-15

**Authors:** Aleksandra Pluta, Michał Jan Stasiowski, Anita Lyssek-Boroń, Seweryn Król, Lech Krawczyk, Ewa Niewiadomska, Jakub Żak, Magdalena Kawka, Dariusz Dobrowolski, Beniamin Oskar Grabarek, Izabela Szumera, Anna Missir, Robert Rejdak, Przemysław Jałowiecki

**Affiliations:** 1 Department of Emergency Medicine, Faculty of Medical Sciences in Zabrze, Medical University of Silesia, 41-200 Sosnowiec, Poland; apluta@autograf.pl (A.P.); lech.kraw@gmail.com (L.K.); sernik7@gmail.com (J.Ż.); iza_sz@vp.pl (I.S.); olaf@pro.onet.pl (P.J.); 2 Department of Anaesthesiology and Intensive Therapy, 5th Regional Hospital, 41-200 Sosnowiec, Poland; seweryn.krol@gmail.com (S.K.); aniami521@interia.pl (A.M.); 3 Department of Ophthalmology with Paediatric Unit, 5th Regional Hospital, 41-200 Sosnowiec, Poland; anitaboron3@gmail.com (A.L.-B.); mkawka.buszman@gmail.com (M.K.); 4 Department of Ophthalmology, Faculty of Medicine in Zabrze, University of Technology in Katowice, 41-800 Zabrze, Poland; 5 Department of General, Colorectal and Polytrauma Surgery, Faculty of Health Sciences in Katowice, Medical University of Silesia, 40-555 Katowice, Poland; 6 Department of Epidemiology and Biostatistics, School of Public Health in Bytom, Medical University of Silesia, 41-902 Bytom, Poland; e.j.niewiadomska@gmail.com; 7 Chair and Clinical Department of Ophthalmology, Faculty of Medical Sciences in Zabrze, Medical University of Silesia, 40-760 Katowice, Poland; dardobmd@wp.pl; 8 Department of Histology, Cytophysiology and Embryology, Faculty of Medicine in Zabrze, University of Technology in Katowice, 41-800 Zabrze, Poland; bgrabarek7@gmail.com; 9 Department of General Ophthalmology, Medical University of Lublin, 20-059 Lublin, Poland; robert.rejdak@umlub.pl

**Keywords:** pars plana vitrectomy (PPV), adequacy of anesthesia (AoA), postoperative nausea and vomiting (PONV), oculo-emetic reflex (OER), oculo-cardiac reflex (OCR)

## Abstract

The intraprocedural immobilization of selected subsets of patients undergoing pars plana vitrectomy (PPV) requires the performance of general anesthesia (GA), which entails the intraoperative use of hypnotics and titration of opioids. The Adequacy of Anesthesia (AoA) concept of GA guidance optimizes the intraoperative dosage of hypnotics and opioids. Pre-emptive analgesia (PA) is added to GA to minimize intraoperative opioid (IO) usage. The current additional analysis evaluated the advantages of PA using either COX-3 inhibitors or regional techniques when added to AoA-guided GA on the rate of presence of postoperative nausea and vomiting (PONV), oculo-emetic (OER), and oculo-cardiac reflex (OCR) in patients undergoing PPV. A total of 176 patients undergoing PPV were randomly allocated into 5 groups: (1) Group GA, including patients who received general anesthesia alone; (2) Group T, including patients who received preventive topical analgesia by triple instillation of 2% proparacaine 15 min before induction of GA; (3) Group PBB, including patients who received PBB; (4) Group M, including patients who received PA using a single dose of 1 g of metamizole; (5) Group P, including patients who received PA using a single dose of 1 g of acetaminophen. The incidence rates of PONV, OCR, and OER were studied as a secondary outcome. Despite the group allocation, intraoperative AoA-guided GA resulted in an overall incidence of PONV in 9%, OCR in 12%, and OER in none of the patients. No statistically significant differences were found between groups regarding the incidence of OCR. PA using COX-3 inhibitors, as compared to that of the T group, resulted in less overall PONV (*p* < 0.05). Conclusions: PA using regional techniques in patients undergoing PPV proved to have no advantage when AoA-guided GA was utilised. We recommend using intraoperative AoA-guided GA to reduce the presence of OCR, and the addition of PA using COX-3 inhibitors to reduce the rate of PONV.

## 1. Introduction

At present, although the performance of regional peribulbar blocks (PBB) in patients undergoing pars plana vitrectomies (PPV) is gaining in gaining popularity [1,2,3], there are still numerous subsets of patients with contraindications to PBB or who require GA for immobilization who fail to co-operate with the operator [4].

The dosing of analgesia and hypnosis during GA, determined by observation of hemodynamic parameters combined with clinical judgement of the anesthesiologist, faced a decline since the Adequacy of Anesthesia concept (AoA) for GA guidance was introduced into everyday practice in many centers [5].

The utility of response and state entropy (RE and SE) in ensuring the proper depth of hypnosis, as volatile anesthetic agents tend to blunt the hemodynamic response to nociceptive stimulation [6] and surgical pleth index (SPI) to determine the proper nociception/antinociception balance [7,8], reduced the cumulative dose of intraoperative opioids (IO) during GA [9], reduced the use of hypnotics, and shortened emergence and length of stay times in the postanesthesia care unit (PACU) [10]. They also decreased the rate of postoperative intolerable pain perception (PIPP) [11].

Additionally, various methods of preventive analgesia (PA) for PPV, such as regional techniques [12,13,14,15,16,17,18,19,20], and intravenous techniques with preoperative infusion of COX-3 inhibitors (paracetamol, metamizole) [21,22], were shown to provide adequate postoperative analgesia [17], with a fall in the rate of main adverse events [12,15,23] within the mechanism of a reduction in demand for IO.

In our previous work, we evaluated the effect of AoA-guided intraoperative titration of IO using fentanyl (FNT) on the dose of the cumulative IO, hemodynamic stability, as efficacy of postoperative analgesia assessed by SPI and NRS values, and in patients undergoing PPV under GA alone or in combination with different regional or intravenous PA techniques [24]. The additional analysis of secondary outcomes aimed to analyze the rate of postoperative nausea and vomiting (PONV), oculo-cardiac reflex (OCR), and oculo-emetic reflex (OER) as perioperative adverse events.

## 2. Materials and Methods

This section builds upon our previous work. Vitrectomies were performed by the same ophthalmic surgeon (AL-B). Details of the procedure were described in an earlier work. Ethical approval for this study (KNW-1-183/N/9/K) was provided by the Ethical Committee of Medical University of Silesia on 29 September 2015 (Chairman: Prof. Maria Trusz-Gluza, MD PhD). The project was registered in the Clinical Trial Registry (SilesianMUKOAiIT2, NCT02973581).

Patients were randomly allocated into five groups: (1) Group GA, including patients who received general anesthesia alone; (2) Group T, including patients who received preventive topical analgesia by triple instillation of 2% proparacaine (alcaine, propacaine hydrochloride ophthalmic solution USP 0.5%, 15 mL, Sandoz a Novartis Company, Warszawa, Poland) 15 min before induction of GA; (3) Group PBB, including patients who received PBB using a mixture of 3.5 mL each of 2% lignocaine (Lignocainum hydrochloricum WZF 2% solution, 20 mg/mL, 2 mL, Polfa Warszawa S.A, Warszawa, Poland), and 0.5% bupivacaine (Bupivacainum hydrochloricum WZF 0.5%, 5 mg/mL, 10 mL, Polfa Warszawa S.A, Warsaw, Poland) with Hamilton’s technique, 1 min before induction of GA [25]; (4) Group M, including patients who received PA using a single dose of 1 g of metamizole (Pyralgin 0.5 g/mL, 5 mL solution; Polpharma SA, Starogard Gdański, Poland) in 100 mL of saline solution intravenously 30 min before arrival at operating room; (5) Group P, including patients who received PA using a single dose of 1 g of acetaminophen (Paracetamol Kabi 10 mg/mL, solution 100 mL; Fresenius Kabi, Kutno, Poland) in 100 mL of saline solution intravenously, 30 min before arrival at the operating room [24].

On the day of surgery, all patients were premedicated with midazolam (Dormicum Midazolam 7.5 mg, Roche Polska Sp z o. o., Warszawa, Poland) 3.75–7.5 mg before induction of anesthesia according to body weight and age [25]. Before the commencement of surgery, patients were preoxygenated for 5 min with 100% oxygen and intravenously infused with 10 mL/kg body weight of Ringer’s lactate solution (500 mL solution, Fresenius Kabi, Kutno, Poland). Anesthesia was induced intravenously with FNT 1 µg/kg body weight (Fentanyl WZF, Fentanyl citrate, 50 microgram/mL, 2 mL solution, Polfa Warszawa S.A, Warszawa, Poland) and etomidate (Etomidate Lipuro, 2 mg/mL, 10 mL, B. Braun Pakistan (Pvt.) Limited, Karachi, Pakistan) 0.2–0.3 mg/kg body weight. After loss of consciousness, rocuronium was administered at a standard intravenous dose of 0.6 mg/kg (Esmeron, rocuronium bromide, 10 mg/mL, 5 mL, Fresenius Kabi, Kutno, Poland) for neuromuscular blockade, followed by the placement of an LMA. The exhaled carbon dioxide concentration (EtCO2) level was maintained at 35–37 mmHg after LMA placement and before the commencement of surgery; the sevoflurane concentration was maintained at a level of approximately 35–45 on state entropy (SE) [24].

Throughout anesthesia induction and surgery, standard monitoring was carried out, paying close attention to vital parameters, including the noninvasive arterial pressure (NIBP), HR, standard ECG lead II, pulse oximetry (SaO_2_), fraction of inspired oxygen in the gas mixture, fraction of inspired sevoflurane (FiAA), fraction of expired sevoflurane, EtCO2, and minimal alveolar concentration of sevoflurane. The depth of anesthesia was monitored with entropy EEG (state and response). Intraoperative analgesia was guided by SPI, and neuromuscular blockade was monitored (Carescape B650, Servsystem, Tyczyn, Poland) [24].

In stage 1, on the admission of patients to the operating theatre, we placed the en-tropy EEG (state and response) sensor on their forehead, a pulse oximeter (SPI) on their finger, contralateral to venous access, an NIBP cuff on their right arm, and standard ECG leads on their back, according to the manufacturer’s suggestions. The baseline values were then recorded.

PBB was performed by the same ophthalmologist (MK), with over 6 years of experience with the procedure, including at least 400 PBBs a year. The sensory block was confirmed based on abolition of the corneal reflex [24].

In stage 2, SPI values were noted from 5 min after LMA placement to the beginning of sterilization of the orbit to calculate the mean SPI value and allow for the calibration of the SPI sensor. The target state and response were around 45.

In stage 3, or the intraoperative stage, the SPI score was monitored online and recorded at 1-min intervals. When an ΔSPI > 15 points above the mean SPI value of stage 2 was reached, a rescue dose of 1 µg/kg of FNT was administered intravenously every 5 min until the SPI value decreased to the mean SPI value in stage 2. The procedure time of VRS was taken as the duration from speculum insertion to removal [24].

We assumed that the initial dose of FNT of 1 µg/kg would produce sufficient an-algesia to insert of the speculum. In addition, Gruenewald et al. [7] proposed ∆SPI > 10 points or an absolute SPI > 50 points as predictors of inadequate analgesia. In our study, we used the protocol of ∆SPI > 15 points compared to the mean value in stage 2, lasting for at least 1 min, as an indication of rescue analgesia. We used this threshold to avoid the possible hazardous overdosing of FNT resulting from potential miscalculations of the SPI value due to its variations [24].

Vitrectomies were performed by the same ophthalmic surgeon (AL-B), with over 10 years of experience with VRS, including over 400 vitreoretinal procedures per year. Eye globe preparation included 3- or 423-Gauge ports [23]. Initial core vitrectomy was followed by peripheral vitreous removal with scleral indentation. The removal of epiretinal membranes or/with internal limiting membrane peeling was the next surgical step. If needed, four types of tamponades were applied: temporary heavy perfluorocarbon liquids, air, SF6 or silicon oil. All retinal brakes or degenerations were treated with laser photocoagulation. The incidence of intraoperative OCR was recorded, typically identified by a rapid 20% decrease in HR from the baseline during ocular manipulations. If OCR occurred, the surgeon was asked to stop surgical stimulation; intravenous atropine 0.5 mg (Atropinum sulfuricum WZF 1 mg/mL, 1ml solution, Polfa Warszawa S.A, Poland) was administered in cases of persisting bradycardia. If persisting hypotension occurred, a single dose of crystalloid was infused intravenously in a dose of 5 mL per kilogram of body weight, and a single dose of 5 mg of ephedrine (Ephedrinum hydrochloricum WZF 25 mg/mL, 1 mL solution, Polfa Warszawa S.A, Poland) was used if crystalloid infusion failed to increase the MAP > 65 mmHg [24].

We assumed that the initial dose of FNT of 1 µg/kg would produce sufficient analgesia to insert of the speculum.

In the postoperative stage, patients were shifted to the postoperative care unit (PACU), and patient monitoring was continued, with the SPI, HR, systolic arterial pressure (SAP), mean arterial pressure (MAP), diastolic arterial pressure (DAP), and SaO_2_ measured by the anesthesiology team blinded to group allocation. Along with postoperative hemodynamic parameters, the presence of adverse effects, such as PONV and allergic reactions, was monitored for each patient. These observations were made at the time of pain assessment for the first 24 h post operation. *Nausea* was defined as subjective sensation of discomfort associated with awareness of the urge to vomit, whereas *Vomiting* was defined as a forceful expulsion of gastric contents through the mouth, as in the report of Abouammoh MA et al. [26]. In cases of PONV, ondansetron 4 mg (Ondansetron Accord, Accord Healthcare Limited, UK) [24] was administered intravenously as the therapy of choice, or as an alternative, to meet the patients’ individual needs, according to contemporary guidelines [27]. Optylite solution 5 mL/kg was infused in cases of MAP < 65 mmHg. Oxygen was administered at a rate of 3 L/min through a nasal cannula. Patients were asked to assess their perception of pain intensity on the numeric pain-rating scale (NRS) ranging from 0 (no pain) to 10 (maximum pain) every 10 min. In the case of an NRS score > 3, nonsteroid anti-inflammatory drugs at the standard dose were administered according to contemporary guidelines for acute pain treatment, issued by the Polish Society of Anesthesiologists [28]. SPI was monitored online, and mean SPI values were recorded at 1-min intervals (trends in software provided by the manufacturer). The NRS and SPI values were recorded for severe (NRS 7–10), moderate (NRS 4–6), and mild pain (NRS 0–3) perception intervals. Patients were observed and monitored in the recovery room for at least 30 min before being transferred to the Department of Ophthalmology when their Aldrette’s score was 10 points [24]. Monitoring and data recording were then ceased. Patients were also evaluated for the risk of PONV using the APFEL score before the procedure to ensure the homogeneity of the groups. Apfel score is a widely recognized risk score, with broad applicability in predicting the incidence of PONV in adults undergoing inhalational anesthesia for various types of surgical procedure. Female gender, history of motion sickness or PONV, nonsmoking status, and the use of postoperative opioids were recognized as constituting risk. The incidences of PONV were estimated as follows: 10%, 21%, 39%, 61%, and 79% if none, one, two, three, or four of the abovementioned independent risk factors were present, respectively [24,29,30].

Early PONV was defined as incidence of PONV observed in the PACU, whereas late PONV was defined as the same incidence observed in the Department of Ophthalmology; overall, PONV was thus defined as incidence of either early and/or late PONV.

### Statistical Analysis

Statistical analysis was performed using the STATISTICA 12PL software (StatSoft Sp. z o.o., Cracow, Poland) with a statistical significance threshold of *p* < 0.05. The Shapiro–Wilk test was used to assess the normality of the distribution. When the assumptions of the normal distribution were met, one-way ANOVA was used to compare more than two variables, followed by Tukey’s posthoc test. On the other hand, when parametric tests could not be used, the Kruskal–Wallis multiple comparison test (nonparametric equivalent of ANOVA) was used, followed by Dunn’s posthoc test. The comparison of the variables on a nominal scale was performed by chi-square analysis.

## 3. Results

Out of the 176 patients allocated to receive AoA-guided GA, 175 patients were analyzed—one patient was excluded due to intraoperative technical problems with SPI monitoring. The anthropometric data of patients who underwent final analysis are shown in the supplementary material (Table S1). 

To ensure the homogeneity of the groups in view of the risk of incidence of PONV, a proper stratification was performed using the Apfel score. No statistically significant differences were noted between groups in terms of both Apfel score and Apfel%. A detailed analysis of separate of different Apfel score dimensions, such as gender, motion sickness, smoking status, and postoperative use of opioid drugs, was also performed. A statistically significant difference was noted regarding history of motion sickness between the M and PBB groups (*p* < 0.05; Table 1).

To estimate the efficacy of the AoA guidance for GA administration, the demand for IO using FNT, as well as fluid challenge, was studied. Significantly higher FNT values were registered among patients from the M group, whereas, regarding fluid challenge, no statistically significant differences were noted (see Table 1). Only 16 (9%) patients declared overall PONV, 8 (23%) patients in the T group, 4 (11%) patients in the GA group, 2 (6%) patients in the PBB group, 1 (3%) patient in the M group and 1 (3%) in the P group; significant differences in percentages between groups M, P, and T were noted. Therefore, further analysis looked at the separate rate of incidence of PONV for early PONV (in the PACU) and late PONV (in the DO). Significant differences were found in percentages between groups M, P, PBB, and T regarding PONV in the PACU, whereas no differences were found regarding PONV in the DO. Only 21 (12%) incidences of OCR were observed; no significant differences were found between groups. No OER event was observed during the study (please see the Table 1).

Further analysis intended to study the overall incidence rate of PONV and OCR, in view of the probability of PONV incidence based on Apfel score analysis, despite the group allocation. No statistically significant differences were found in this aspect (see Table 2).

Further in-depth analysis was performed to investigate the correlation between the rate of incidence of overall PONV, despite group allocation, according to the number of separate risk factors for the incidence of PONV, expressed via the Apfel score. There was a statistically significant increase in patients at risk of the presence of 1 and 2 separate risk factors, compared to 0 and 3 risk factors. Nevertheless, we found no statistically significant difference in the incidence of overall PONV, despite the number of risk factors for PONV. The comparison of the variables on a nominal scale was performed by chi-square analysis with pairwise comparisons of proportions (Holm); please see the Table 3.

## 4. Discussion

Regardless of the type of operation, the general incidence of vomiting is estimated at about 30%, incidence of nausea at about 50%, and, in subgroups of high-risk patients, the PONV rate can be as high as 80% [31,32,33,34]. 

Carvalho et al. [35] observed a lower incidence of PONV in patients undergoing mechanical vitrectomy under a regional block as compared to GA. Additionally, total intravenous anesthesia using propofol and alfentanil was observed to correlate with a reduction in PONV incidence rate to 16% from 43.5% [35], as compared to that of balanced anesthesia in the observations of Heinke et al. [36]. 

In all these studies, IO, either inhalational anesthetics or hypnotic drugs administration, was presented based on the observation of hemodynamic fluctuations and the anesthesiologist’s judgement, whereas we adopted AoA guidance, for the first time, for patients undergoing PPV. A statistically significant reduction in the incidence of overall PONV was found between the T group and both groups with PA using COX-3 inhibitors. The 3% PONV incidence rate among patients receiving COX-3 inhibitors was quite an achievement when compared to that of the abovementioned literature, even in those underlining the superiority of PBB with monitored anesthesia care regarding the reduction in PONV incidence rate after PPV.

In the current study, although late PONV was not statistically significant (in the DO) between the studied groups, no incidence of late PONV was observed in the P and M group, which is also noteworthy in view of the quality of postoperative care.

To reduce the PONV incidence rate, numerous preventive anesthetic regimens were employed, mainly based on preoperative antiemetic pharmacological premedication. Reibaldi et al. [37], in their study concerning PPV under PBB, based their regimen on the administration of either ondansetron or dexamethasone, or used a multimodal approach based on both, as compared to that of a placebo [37].

In the current study, although no preventive antiemetic regimen was introduced, the PONV incidence rate was lower as compared to that of the literature data. Our results demonstrated that the appropriate administration of intraoperative inhalational anesthetic and rescue IO under AoA guidance might prevent an excessive IO dosage, resulting in a lower incidence of PONV compared to that of the abovementioned literature data. Thus, we suggest that the precision of the intraoperative administration of IO leads not only to stable hemodynamics and a presence of unacceptable postoperative pain perception, as presented in our former report [24], but could possibly constitute an emerging alternative to the utility of a preventive antiemetic regimen, which was reported to be responsible for the rare but life-threatening complications associated with their use [38,39,40] in patients with no or one risk factor for PONV.

The anesthetic regimen based on GA using IO and inhalational anesthetics is widely reported to predispose the incidence of PONV; therefore, an Apfel score dedicated to patients who were anesthetized using inhalational anesthetics was calculated to ensure homogeneity regarding the probability of PONV incidence [41,42]. To the best of our knowledge on the literature in this province, such a stratification was never performed before among patients undergoing PPV under GA. 

We hypothesized that, in the current study, AoA guidance eliminated the influence of independent PONV risk factors on the incidence of PONV. Nevertheless, among patients with 2 and 3 independent risk factors, the PONV rate was 11.1% and 13.3%, respectively, whereas in the observation of Heinke et al. [36], the addition of preventive antiemetic prophylaxis to GA with PBB further reduced the incidence of PONV to 4.7%. 

Interestingly, the employment of regional anesthesia techniques in the current study did not prove to be useful when compared to the GA group, contrary to the majority of the abovementioned literature. Similar findings were obtained regarding hemodynamic stability and PIPP incidence in our preliminary report [43,44,45,46].

On the one hand, the demand for rescue IO was the highest among patients receiving metamizole, and the highest percentage of patients with motion sickness who were predisposed to PONV were found in this group. On the other hand, the incidence rate of PONV was the lowest among this group. The demand for rescue IO was similarly low for patients receiving paracetamol, as well as for patients from the PBB group, using a mixture of lidocaine and bupivacaine.

In the current study, we suppose that the precision of administration of inhalational anesthetic with an SE target value of around 45, alongside the precision of rescue IO administration with target delta SPI 15, supposedly produced a stable enough GA that intraoperative pressure on the eyeball and/or traction of extraocular muscles did not result in an HR decrease, resulting in hemodynamic instability.

Asmer et al. [47], who achieved OCR incidence rates in similar groups to the current study, observed that the combination of GA and sub-Tenon’s block, using levobupivacaine in retinal surgery patients, decreases the incidence of OCR to 3 cases out of 40, as compared to that of general anesthesia alone, which decreases OCR incidence to 6 cases out of 40 [43]. No statistically significant differences were observed between groups in the current study, so even AoA guidance for GA eliminated the hypothetical benefit of PA using different techniques, especially regional anesthesia techniques (PBB, TA), which were reported to reduce the incidence of OCR in the literature. Thus, we assume that the key to a reduction in the OCR incidence rate may not necessarily be caused by the addition of PA, especially using regional techniques, but by stable hypnosis and analgesia of GA under AoA guidance, regardless of the technique used. It is possible that the AoA guidance in the current study, with or without the addition of PBB or TA, produced a similarly low incidence of OCR to the addition of PBB or a sub-Tenons block to GA without AoA guidance for GA, as in the literature.

OER was first described by Van den Berg A.A. et al., who observed the OCR produced by squint surgery in children. They hypothesized that there was an association between the occurrence of OCR and PONV. The high incidence of PONV in their study, particularly the early PONV associated only with squint surgery, producing intraoperative OCR, provided clinical evidence for the existence of OER [48]. In their further studies they described the afferent pathway of the OER, sharing its afferent limb of the reflex arc, with the OCR [49]. Again, in our view, the adequate administration of intraoperative IO using AoA guidance and stable SE guidance for inhalational anesthetic agent administration could lead to a stable deep GA and, therefore, result in a low incidence of OCR and PONV, as compared to data from the literature. 

Premedication using midazolam was proven to decrease the rate PONV after VRS, which was a part of the anesthetic regimen, despite the group allocation. Therefore, its administration possibly proportionally affected the final results. Secondly, nausea can be perceived as a subjective phenomenon, which may be underreported by patients. Thirdly, the current literature provides no consistent algorithm for titration of rescue opioid analgesics based on the online observation of fluctuations in SPI value [50].

Therefore, similarly to our previous studies, we adopted a methodology in which the intraprocedural increase in SPI value by 15, from the baseline calculated during the calibration of an SPI sensor, between LMA installation and start of the VRS (stage 2), constituted an indication to administer a single rescue dose of intravenous FNT [51,52]. Finally, an in-depth analysis of the risk factors for the occurrence of PONV and OCR in patients with certain comorbidities undergoing PPV under AoA-guided GA will be published separately due to the word count limit, vast volume of collected data, and complexity of analysis.

## 5. Conclusions

In patients undergoing PPV, we recommend AoA guidance of GA to ensure the equal depth of anesthesia through the administration of inhalational hypnotics, as reflected in the SE value, and proper dosage of IO, as reflected in the SPI value. Together, these guarantee intraoperative stability, limiting the OCR incidence rate and risk to hemodynamic stability, which has possible life-threatening consequences. Additionally, we recommend no addition of regional techniques such as topical anesthesia and PBB using bupivacaine and lidocaine mixture because their use may lead to unwelcome complications, take more time, and impair economic frugality. Furthermore, no benefit to their use was observed as compared to that of the GA group. Moreover, we recommend the addition of COX-3 inhibitors to AoA-guided GA because such an anesthetic regimen resulted in the lowest rate of PONV, despite the varied dose of IO required for stable GA. Further studies, especially multicenter studies, which we would gladly participate in, are required to investigate the possibility of the complete eradication of PONV and further reductions in OCR incidence based on the employment of a combination of already available anesthetic techniques, possibly combining AoA guidance for GA with antiemetic prophylaxis based on the pre-procedural calculation of Apfel score, so that antiemetic prophylaxis can be adjusted accordingly.

## Figures and Tables

**Table 1:** Rate of PONV, OCR, and OER in patients regarding group allocation.

Intraoperative Data	Total *n* = 175 (100)	GA Group *n* = 35 (20)	M Group *n* = 35 (20)	P Group *n* = 35 (20)	PBB Group *n* = 35 (20)	T Group *n* = 35 (20)	*p*-Value
X ± S/M (Rk)							
FNT requirement (mg) (IO)	129.9 ± 108.2 100 (150)	144.3 ± 102.7 150 (150)	165.7 ± 116.8 200 (200)	95.7 ± 81.7 100 (50)	95.1 ± 101.3 50 (150)	148.6 ± 120.3 150 (200)	*p* < 0.05 ^A^
Intraoperative fluid challenge	991.8 ± 303.6 1000 (350)	955.7 ± 376.1 1000 (450)	1014.3 ± 334.4 1000 (500)	926.7 ± 196.4 1000 (250)	1075.7 ± 281.6 1000 (250)	977.4 ± 282.3 1000 (350)	*p* = 0.16 NS
No/yes *n* (%)							
Overall PONV	159 (91)/16 (9)	31 (89)/4 (11)	34 (97)/1 (3)	34 (97)/1 (3)	33 (94)/2 (6)	27 (77)/8 (23)	*p* < 0.05 ^B^
PONV in the PACU	161 (92)/14 (8)	31 (88.6)/4 (11.4)	34 (97.1)/1 (2.9)	34 (97.1)/1 (2.9)	34 (97.1)/1 (2.9)	28 (80)/7 (20)	*p* < 0.05 ^C^
Nausea in the PACU	169 (97)/6 (3)	35 (100)/0 (0)	34 (97)/1 (3)	34 (97)/1 (3)	35 (100)/0 (0)	31 (89)/4 (11)	*p* = 0.06 NS
Vomiting in the PACU	167 (95)/8 (5)	31 (89)/4 (11)	35 (100)/0 (0)	35 (100)/0 (0)	34 (97)/1 (3)	32 (91)/3 (9)	*p* = 0.07 NS
PONV in the DO	166 (95)/9 (5)	34 (97)/1 (3)	35 (100)/0 (0)	35 (100)/0 (0)	31 (89)/4 (11)	31 (89)/4 (11)	*p* = 0.05
Nausea in the DO	172 (98)/3 (2)	35 (100)/0 (0)	35 (100)/0 (0)	35 (100)/0 (0)	33 (94)/2 (6)	34 (97)/1 (3)	*p* = 0.24 NS
Vomiting in the DO	169 (97)/6 (3)	34 (97)/1 (3)	35 (100)/0 (0)	35 (100)/0 (0)	33 (94)/2 (6)	32 (91)/3 (9)	*p* = 0.12 NS
PONV in the PACU and DO	172 (98)/3 (2)	34 (97)/1 (3)	35 (100)/0 (0)	35 (100)/0 (0)	34 (97)/1 (3)	34 (97)/1 (3)	*p* = 0.73 NS
Nausea in the PACU and DO	174 (99)/1 (1)	35 (100)/0 (0)	35 (100)/0 (0)	35 (100)/0 (0)	35 (100)/0 (0)	34 (97)/1 (3)	*p* = 0.52 NS
Vomiting in the PACU and DO	173 (99)/2 (1)	34 (97)/1 (3)	35 (100)/0 (0)	35 (100)/0 (0)	34 (97)/1 (3)	35 (100)/0 (0)	*p* = 0.45 NS
OCR	154 (88)/21 (12)	31 (88.6)/4 (11.4)	29 (82.9)/6 (17.1)	31 (88.6)/4 (11.4)	31 (88.6)/4 (11.4)	32 (91.4)/3 (8.6)	*p* = 0.87 NS
OER	175 (100)/0 (0)	35 (100)/0 (0)	35 (100)/0 (0)	35 (100)/0 (0)	35 (100)/0 (0)	35 (100)/0 (0)	-
APFEL score	1.5 ± 0.7 2 (1)	1.5 ± 0.7 1 (1)	1.5 ± 0.9 1 (1)	1.7 ± 0.5 2 (1)	1.4 ± 0.6 1 (1)	1.4 ± 0.6 1 (1)	*p* = 0.3 NS
APFEL (%)	0.3 ± 0.1 0.4 (0.2)	0.3 ± 0.1 0.2 (0.2)	0.3 ± 0.1 0.2 (0.2)	0.3 ± 0.1 0.4 (0.2)	0.3 ± 0.1 0.2 (0.2)	0.3 ± 0.1 0.2 (0.2)	*p* = 0.3
Gender Female/male	97 (55.4)/ 78 (44.6)	18 (51.4)/ 17 (48.6)	15 (42.9)/ 20 (57.1)	24 (68.6)/ 11 (31.4)	21 (60)/ 14 (40)	19 (54.3)/ 16 (45.7)	*p* = 0.26
Motion sickness No/yes	161 (92)/ 14 (8)	33 (94)/ 2 (6)	28 (80)/ 7 (20)	32 (91)/ 3 (9)	35 (100)/ 0 (0)	33 (94)/ 2 (6)	*p* < 0.05
Smoking No/yes	152 (87)/ 23 (13)	31 (89)/ 4 (11)	30 (86)/ 5 (14)	33 (94)/ 2 (6)	29 (83)/ 6 (17)	29 (83)/ 6 (17)	*p* = 0.54
Postoperative use of opioid drugs No/yes	175 (100)/ 0 (0)	35 (100)/ 0 (0)	35 (100)/ 0 (0)	35 (100)/ 0 (0)	35 (100)/ 0 (0)	35 (100)/ 0 (0)	-

Results presented as mean ± SD and median (IQR) for quantitative variables and numbers (percentages) for nominal variables. *p*-values by one-way ANOVA test for quantitative variables; *p*-values by posthoc tests: ^A^ significantly less in Group PBB than in Group M (*p* < 0.05); *p*-values by X2 test for nominal variables; pairwise comparison of proportions; ^B^ significant differences in percentages between groups M and P; ^C^ significant differences in percentages between groups PBB and T. Abbreviations: group GA—patients who received general anesthesia; group M—patients who received PA using a single dose of 1 g of metamizole intravenously, 30 min before arrival at operating room; group P—patients who received PA using a single dose of 1 g of acetaminophen intravenously, 30 min before arrival at the operating room; group PBB—including patients who received PBB using a mixture of 3.5 mL each of 2% lignocaine and 0.5% bupivacaine with Hamilton’s technique, 1 min before induction of GA; group T—patients who received preventive topical analgesia by triple instillation of 2% proparacaine; FNT—fentanyl, IO—intraoperative opioids, PONV—postoperative nausea and vomiting; PACU—postanesthesia care unit, DO—Department of Ophthalmology, OCR—oculocardiac reflex; OER—oculoemetic reflex, SD—standard deviation; IQR—interquartile range; BMI—body mass index.

**Table 2:** Apfel score in patients according to presence of PONV and OCR.

Scale *n* = 175 (100)	Overall PONV *n* = 16 (100)	No-PONV *n* = 159 (100)	*p*-Value	OCR *n* = 21 (100)	No-OCR *n* = 154 (100)	*p*-Value
APFEL score	1.8 ± 0.5 2 (0.5)	1.5 ± 0.7 1 (1)	*p* = 0.07 NS	1.5 ± 0.5 2 (1)	1.5 ± 0.7 2 (1)	*p* = 0.93 NS
APFEL (%)	0.4 ± 0.1 0.4 (0.1)	0.3 ± 0.1 0.2 (0.2)	*p* = 0.07 NS	0.3 ± 0.1 0.4 (0.2)	0.3 ± 0.1 0.4 (0.2)	*p* = 0.93 NS

Results presented as mean ± SD and median (IQR) for quantitative variables; *p*-values by the Mann–Whitney U test for quantitative variables. Abbreviations: PONV—postoperative nausea and vomiting; OCR—oculocardiac reflex.

**Table 3:** Apfel score of patients according to postoperative presence or absence of PONV.

APFEL Score [Point] *n* (%)	0 (10% Risk of PONV)	1 (21% Risk of PONV)	2 (39% Risk of PONV)	3 (61% Risk of PONV)	*p*-Value
Total *n* = 175 (100)	10 (5.7)	74 (42.3)	84 (48)	7 (4)	*p* < 0.0001 ^A^
PONV	0 (0)	4 (5.4)	11 (13.1)	1 (14.3)	*p* = 0.26 NS
No-PONV	10 (100)	70 (94.6%)	73 (86.9)	6 (85.7)	*p* = 0.26 NS

Results presented as numbers (percentages) for nominal variables; *p*-values by the test for equality of proportions for nominal variables. Pairwise comparison of proportions: ^A^ significant differences in percentages between the groups of 0, 3 and 1, 2 points of Apfel score. Abbreviations: PONV—postoperative nausea and vomiting.

## Data Availability

The data used to support the findings of this study are included within the article.

## Supplementary Materials

The following are available online at https://www.mdpi.com/article/10.3390/jcm10184172/s1, Table S1. Anthropometric data of patients in studied groups. Table modified from our previous work.

## References

[1] Gioia L., Fanelli G., Casati A., Nuti U., Mennella R., Scarioni M., Cerchierini E., Sciascia A., Garassino A., Torri G. (2004). A prospective, randomized, double-blinded comparison of ropivacaine 0.5%, 0.75%, and 1% ropivacaine for peribulbar block. J. Clin. Anesth..

[2] Luchetti M., Magni G., A Marraro G. (2000). A prospective randomized double-blinded controlled study of ropivacaine 0.75% versus bupivacaine 0.5%–mepivacaine 2% for peribulbar anesthesia. Reg. Anesth. Pain Med..

[3] Zhou Y.-L., Tong Y., Wang Y.-X., Zhao P.-Q., Wang Z.-Y. (2017). A prospective, randomised, double-masked comparison of local anaesthetic agents for vitrectomy. Br. J. Ophthalmol..

[4] Fudickar A., Gruenewald M., Fudickar B., Hill M., Wallenfang M., Hüllemann J., Voss D., Caliebe A., Roider J.B., Steinfath M. (2017). Immobilization during anesthesia for vitrectomy using a laryngeal mask without neuromuscular blockade versus endotracheal intubation and neuromuscular blockade. Minerva Anestesiol..

[5] Van Gils M., Korhonen I., Yli-Hankala A. (2002). Methods for Assessing Adequacy of Anesthesia. Crit. Rev. Biomed. Eng..

[6] Bharti N., Chari P., Kumar P. (2012). Effect of sevoflurane versus propofol-based anesthesia on the hemodynamic response and recovery characteristics in patients undergoing microlaryngeal surgery. Saudi J. Anaesth..

[7] Gruenewald M., Ilies C. (2013). Monitoring the nociception–anti-nociception balance. Best Pract. Res. Clin. Anaesthesiol..

[8] Kallio H., Lindberg L.I., Majander A.S., Uutela K.H., Niskanen M.L., Paloheimo M.P.J. (2008). Measurement of surgical stress in anaesthetized children. Br. J. Anaesth..

[9] Bergmann I., Göhner A., Crozier T.A., Hesjedal B., Wiese C.H., Popov A.F., Bauer M., Hinz J.M. (2013). Surgical pleth index-guided remifentanil administration reduces remifentanil and propofol consumption and shortens recovery times in outpatient anaesthesia. Br. J. Anaesth..

[10] Gruenewald M., Harju J., Preckel B., Molnár Z., Yli-Hankala A., Rosskopf F., Koers L., Orban A., Bein B. (2021). Comparison of adequacy of anaesthesia monitoring with standard clinical practice monitoring during routine general anaesthesia. Eur. J. Anaesthesiol..

[11] Upton H.D., Ludbrook G.L., Wing A., Sleigh J.W. (2017). Intraoperative “Analgesia Nociception Index”–Guided Fentanyl Administration During Sevoflurane Anesthesia in Lumbar Discectomy and Laminectomy: A Randomized Clinical Trial. Anesthesia Analg..

[12] Ghali A.M., El Btarny A.M. (2010). The effect on outcome of peribulbar anaesthesia in conjunction with general anesthesia for vitreoretinal surgery. Anaesthesia.

[13] Kolny M., Stasiowski M.J., Zuber M., Marciniak R., Chabierska E., Pluta A., Jałowiecki P., Byrczek T. (2017). Randomized, comparative study of the effectiveness of three different techniques of interscalene brachial plexus block using 0.5% ropivacaine for shoulder arthroscopy. Anaesthesiol. Intensive Ther..

[14] Schönfeld C.-L., Hierneis S., Kampik A. (2012). Preemptive Analgesia with Ropivacaine for Pars Plana Vitrectomy. Retina.

[15] Williams N., Strunin A., Heriot W. (1995). Pain and Vomiting after Vitreoretinal Surgery: A Potential Role for Local Anaesthesia. Anaesth. Intensive Care.

[16] Bayerl K., Boost K., Wolf A., Kampik A., Schaumberger M., Haritoglou C. (2014). 23-Gauge-Pars-plana-Vitrektomien in Allgemeinanästhesie. Der Ophthalmol..

[17] Henzler D., Müller-Kaulen B., Steinhorst U.H., Broermann H., Piepenbrock S. (2002). Die Kombination von retrobulbärer Leitungsanästhesie und Allgemeinanästhesie führt zu präemptiver Analgesie bei Pars-plana-Vitrektomien. AINS Anästhesiol. Intensivmed. Notfallmedizin Schmerzther..

[18] Mahfouz A.K.M., Nabawi K.S. (2002). Preemptive Analgesia in Rhegmatogenous Retinal Detachment Surgery. Retina.

[19] Mason J.O., Goodwin P.L., Feist R.M., Vail R.S. (2007). Preemptive Sub-Tenon’s Anesthesia for Pars Plana Vitrectomy Under General Anesthesia: Is It Effective?. Ophthalmic Surg. Lasers Imaging Retin..

[20] Fraunfelder R. (2009). Safety, efficacy, and patient acceptability of lidocaine hydrochloride ophthalmic gel as a topical ocular anesthetic for use in ophthalmic procedures. Clin. Ophthalmol..

[21] Landwehr S., Kiencke P., Giesecke T., Eggert D., Thumann G., Kampe S. (2005). A comparison between IV paracetamol and IV metamizol for postoperative analgesia after retinal surgery. Curr. Med. Res. Opin..

[22] Sadrolsadat S.H., Yousefshahi F., Ostadalipour A., Mohammadi F.Z., Makarem J. (2017). Effect of Intravenous Acetaminophen on Postoperative Pain in Vitrectomy: A Randomized, Double-Blind, Clinical Trial. Anesthesiol. Pain Med..

[23] Ruta U., Möllhoff T., Markodimitrakis H., Brodner G. (1996). Attenuation of the oculocardiac reflex after topically applied lignocaine during surgery for strabismus in children. Eur. J. Anaesthesiol..

[24] Stasiowski M., Pluta A., Lyssek-Boroń A., Kawka M., Krawczyk L., Niewiadomska E., Dobrowolski D., Rejdak R., Król S., Żak J. (2021). Preventive Analgesia, Hemodynamic Stability, and Pain in Vitreoretinal Surgery. Medicina.

[25] Hamilton R.C. (1995). Techniques of orbital regional anaesthesia. Br. J. Anaesth..

[26] Kusza K., Kübler A., Maciejewski D., Mikstacki A., Owczuk R., Wujtewicz M., Piechota M. (2013). Guidelines of the Polish Society of Anaesthesiology and Intensive Therapy determining principles, conditions and organisational aspects of anaesthesiology and intensive therapy services. Anaesthesiol. Intensive Ther..

[27] Abouammoh M.A., Abdelhalim A.A., Mohamed E.A., Elzoughari I., Mustafa M., Al-Zahrani T.A. (2016). Subtenon block combined with general anesthesia for vitreoretinal surgery improves postoperative analgesia in adult: A randomized controlled trial. J. Clin. Anesth..

[28] Gan T.J., Diemunsch P., Habib A., Kovac A., Kranke P., Meyer T.A., Watcha M., Chung F., Angus S., Apfel C.C. (2014). Consensus Guidelines for the Management of Postoperative Nausea and Vomiting. Anesth. Analg..

[29] Misiołek H., Cettler M., Woroń J., Wordliczek J., Dobrogowski J., Mayzner-Zawadzka E. (2014). Zalecenia postępowania w bólu pooperacyjnym—2014. Anestezjol. Intensywna Ter..

[30] Apfel C.C., Läärä E., Koivuranta M., Greim C.-A., Roewer N. (1999). A Simplified Risk Score for Predicting Postoperative Nausea and Vomiting. J. Am. Soc. Anesthesiol..

[31] Darvall J., Handscombe M., Maat B., So K., Suganthirakumar A., Leslie K. (2021). Interpretation of the four risk factors for postoperative nausea and vomiting in the Apfel simplified risk score: An analysis of published studies. Can. J. Anesth..

[32] Koivuranta M., Läärä E., Snåre L., Alahuhta S. (1997). A survey of postoperative nausea and vomiting. Anaesthesia.

[33] Gan T.J., Belani K.G., Bergese S., Chung F., Diemunsch P., Habib A.S., Jin Z., Kovac A.L., Meyer T.A., Urman R.D. (2020). Fourth Consensus Guidelines for the Management of Postoperative Nausea and Vomiting. Anesth. Analg..

[34] Gan T.J., Sloan F., Dear G.D.L., El-Moalem H.E., Lubarsky D.A. (2001). How Much Are Patients Willing to Pay to Avoid Postoperative Nausea and Vomiting?. Anesth. Analg..

[35] Carvalho B., Jantarada C., Azevedo J., Maia P., Guimarães L. (2020). Comparison of peribulbar block and general anaesthesia in mechanical vitrectomy: A prospective observational study. Rev. Española Anestesiol. Reanim..

[36] Heinke W., Frank T., Meier P., Wiegel M., Korth D. (1996). Postoperative vomiting after pars plana vitrectomy. Anaesthesiol. und Reanim..

[37] Reibaldi M., Fallico M., Longo A., Avitabile T., Astuto M., Murabito P., Minardi C., Bonfiglio V., Boscia F., Furino C. (2019). Efficacy of Three Different Prophylactic Treatments for Postoperative Nausea and Vomiting after Vitrectomy: A Randomized Clinical Trial. J. Clin. Med..

[38] Goodin S., Cunningham R. (2002). 5-HT 3 -Receptor Antagonists for the Treatment of Nausea and Vomiting: A Reappraisal of Their Side-Effect Profile. Oncologist.

[39] Food and Drug Administration FDA Drug Safety Communication: New Information Regarding QT Prolongation with Ondansetron (Zofran). https://www.fda.gov/drugs/drug-safety-and-availability/fda-drug-safety-communication-new-information-regarding-qt-prolongation-ondansetron-zofran.

[40] A Doggrell S., Hancox J.C. (2013). Cardiac safety concerns for ondansetron, an antiemetic commonly used for nausea linked to cancer treatment and following anaesthesia. Expert Opin. Drug Saf..

[41] Pluta M., Krzych L. (2018). Usefulness of Apfel score to predict postoperative nausea and vomiting—Single-center experiences. Ann. Acad. Med. Silesiensis.

[42] Apfel C., Heidrich F., Jukar-Rao S., Jalota L., Hornuss C., Whelan R., Zhang K., Cakmakkaya O. (2012). Evidence-based analysis of risk factors for postoperative nausea and vomiting. Br. J. Anaesth..

[43] Jaichandran V.V., Nair A.G., Gandhi R.A., Prateeba-Devi N. (2013). Brainstem anesthesia presenting as contralateral third nerve palsy following peribulbar anesthesia for cataract surgery. Acta Anaesthesiol. Taiwanica.

[44] Chhabra A., Singh P.M., Kumar M. (2012). Pulmonary oedema in a patient undergoing vitreo-retinal surgery under peribulbar block. Indian J. Anaesth..

[45] Bensghir M., Badou N., Houba A., Balkhi H., Haimeur C., Azendour H. (2014). Convulsions during cataract surgery under peribulbar anesthesia: A case report. J. Med. Case Rep..

[46] Krilis M., Zeldovich A., Garrick R., Goldberg I. (2013). Vision loss and partial third nerve palsy following contralateral peribulbar anesthesia. J. Cataract. Refract. Surg..

[47] Amer G.F., Abdeldayem O.T., Abdulla M.G. (2019). Postoperative analgesic efficacy of sub-Tenon’s block with levobupivacaine in retinal surgery under general anesthesia. Anesth. Essays Res..

[48] Berg A.A., Lambourne A., Yazji N.S., Laghari N.A. (1987). Vomiting after ophthalmic surgery. Anaesthesia.

[49] Berg A.A., Lambourne A., Clyburn P.A. (1989). The oculo-emetic reflex. Anaesthesia.

[50] Ledowski T., Burke J., Hruby J. (2016). Surgical pleth index: Prediction of postoperative pain and influence of arousal. Br. J. Anaesth..

[51] Stasiowski M., Starzewska M., Niewiadomska E., Król S., Marczak K., Żak J., Pluta A., Eszyk J., Grabarek B., Szumera I. (2021). Adequacy of Anesthesia Guidance for Colonoscopy Procedures. Pharmaceuticals.

[52] Stasiowski M., Missir A., Pluta A., Szumera I., Stasiak M., Szopa W., Błaszczyk B., Możdżyński B., Majchrzak K., Tymowski M. (2020). Influence of infiltration anaesthesia on perioperative outcomes following lumbar discectomy under surgical pleth index-guided general anaesthesia: A preliminary report from a randomised controlled prospective trial. Adv. Med. Sci..

