# Supplementary material for: Adverse Events during Vitrectomy under Adequacy of Anesthesia—An Additional Report
Source: J Clin Med. 2021 Sep 15;10(18):4172. doi: 10.3390/jcm10184172 (PMC8468095; doi:10.3390/jcm10184172)
Supplement: Supplementary file 1 [file jcm-10-04172-s001.zip › jcm-1365177-supplementary.pdf]

Table S1. Anthropometric data of patients in studied groups. Table modified from our previous work [24].

Anthropometric Data		Total N = 175 (100)	GA Group N = 35 (20)	M Group N = 35 (20)	P Group N = 35 (20)	PBB Group N = 35 (20)	T Group N = 35 (20)	p-Value
Age X ± S/M (R_k)	[years]	64.5 ± 11.7<br>66 (13)	65.1 ± 10.8<br>67 (9)	61.9 ± 11.9<br>63 (14)	66.1 ± 9.9<br>67 (8)	66.8 ± 12.1<br>69 (13)	62.7 ± 13.3<br>65 (14)	p = 0.25<br>NS
Gender n (%)	female	97 (55.4)	18 (51.4)	15 (42.9)	24 (68.6)	21 (60)	19 (54.3)	p = 0.26<br>NS
	male	78 (44.6)	17 (48.6)	20 (57.1)	11 (31.4)	14 (40)	16 (45.7)	
Height X ± S/M (R_k)	[cm]	165.8 ± 8.7<br>165 (12)	166.9 ± 8.6<br>168 (14)	168 ± 7.4<br>170 (14)	163.4 ± 8.7<br>160 (12)	165.9 ± 8.3<br>164 (12)	164.7 ± 10.3<br>164 (18)	p = 0.18<br>NS
Weight X ± S/M (R_k)	[kg]	77.6 ± 15.9<br>75.5 (17)	83.4 ± 19.8<br>82 (20)	74.7 ± 14.9<br>74 (19)	74.1 ± 13.3<br>74 (22)	78.8 ± 16<br>75 (11)	77.1 ± 13.7<br>80 (21)	p = 0.19<br>NS
BMI X ± S/M (R_k)	[kg/m^2]	28.3 ± 5.4<br>27.5 (6.4)	29.9 ± 6.6<br>28.4 (5.3)	26.4 ± 4.6<br>25.3 (5.4)	27.9 ± 5.3<br>27.6 (7.7)	28.6 ± 5.1<br>27.1 (4.4)	28.5 ± 4.9<br>28.4 (7.3)	p = 0.05
BMI n (%)	norm	50 (28.7)	5 (14.3)	15 (42.9)	14 (40)	7 (20)	9 (25.7)	$p < 0.05$ [A]
	overweight	72 (41.4)	18 (51.4)	13 (37.1)	9 (25.7)	19 (55.9)	13 (37.1)	p = 0.09<br>NS
	obesity	52 (29.9)	12 (34.3)	7 (20)	12 (34.3)	8 (23.5)	13 (37.1)	p = 0.41<br>NS
ASA scale	I	14 (8)	2 (5.71)	2 (5.71)	5 (14.28)	1 (2.86)	4 (11.43)	p = 0.983
	II	122 (69.8)	25 (71.43)	25 (71.43)	21 (60)	26 (74.29)	25 (71.43)	
	III	39 (22.2)	8 (22.56)	8 (22.56)	9 (25.71)	8 (22.56)	6 (17.14)	

Results presented as mean ± SD and median (IQR) for quantitative variables and numbers (percentages) for nominal variables. p-values by the one-way ANOVA test for quantitative variables; p-values by the test for equality of proportions for nominal variables. Pairwise comparison of proportions: [A] Significant differences in percentages between the groups GA and M, P; Abbreviations: group GA—patients who received general anaesthesia; group M—patients who received PA using a single dose of 1 g of metamizole intravenously, 30 min before arrival at operating room; Group P—patients who received PA using a single dose of 1 g of acetaminophen intravenously, 30 min before arrival at operating room; Group PBB—including patients who received PBB using a mixture of 3.5 mL each of 2% lignocaine and 0.5% bupivacaine with Hamilton's technique, 1 min before induction of GA; Group T—patients who received preventive topical analgesia by triple instillation of 2% proparacaine; BMI—body mass index; ASA scale—American Society of Anaesthesiologists scale reflecting general patients' health condition, SD—standard deviation; IQR—interquartile range; NS – non-statistically significance ($p > 0.05$).
